# Supplementary material for: A yeast phenomic model for the influence of Warburg metabolism on genetic buffering of doxorubicin
Source: Cancer Metab. 2019 Oct 23;7:9. doi: 10.1186/s40170-019-0201-3 (PMC6806529; doi:10.1186/s40170-019-0201-3)
Supplement: Supplementary file 9 — Additional file 9. GO term-specific heatmaps for REMc/GTF-enriched clusters. GO term-specific heatmaps for significant GO process terms were generated as described in methods and Figs. 3 and 4. Any related child terms are presented in subsequent pages of the parent file name. GO terms with more than 100 children, with 2 or fewer genes annotated to the term, or a file size over 300KB are not shown. All heatmaps are generated with the same layout (see Figs. 3 and 4). [file 40170_2019_201_MOESM9_ESM.bz2 › Additional_File9_GOTermHeatmaps/Additional_File9_GOTermHeatmap/regulation_of_gene_silencing.pdf]

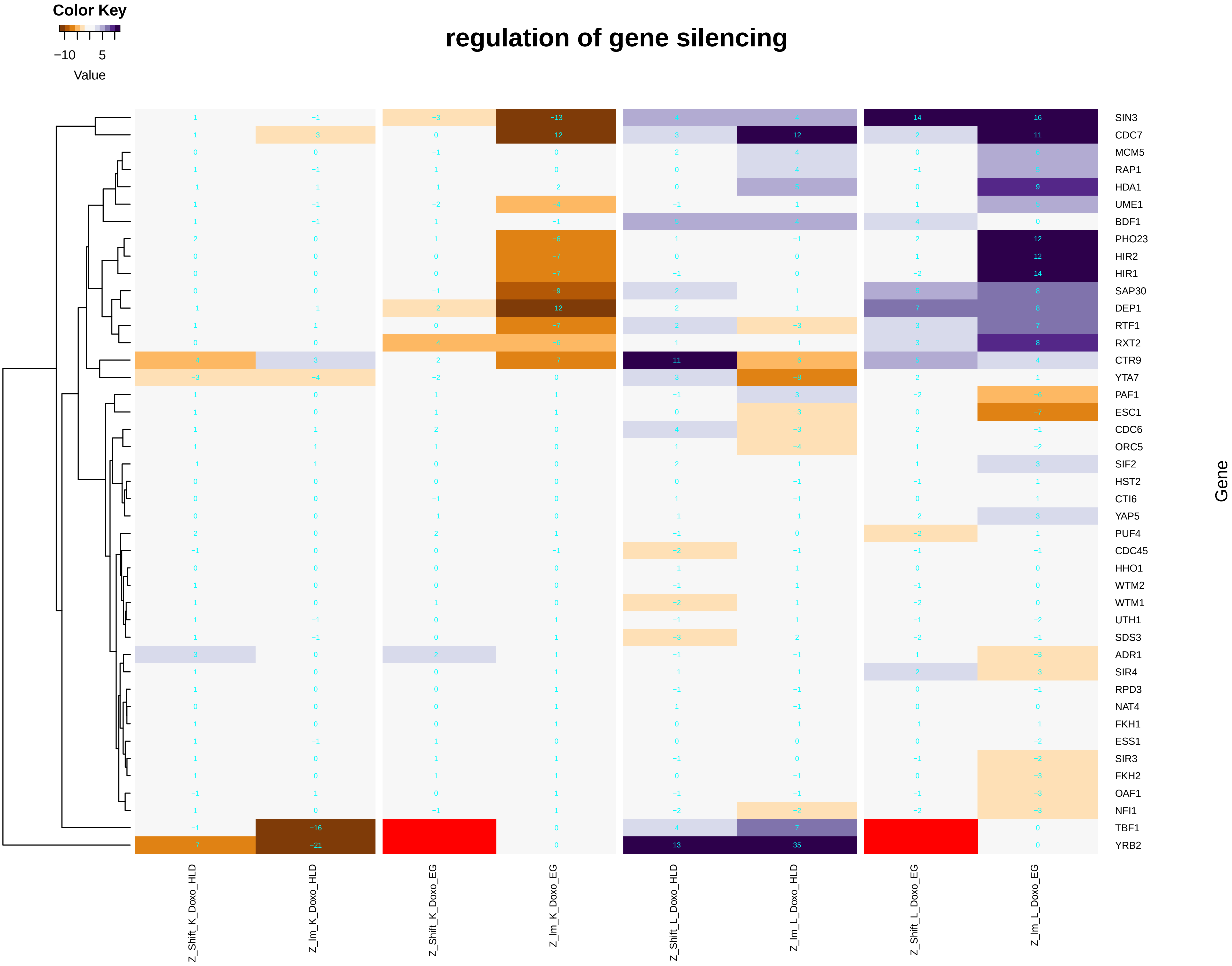
Color Key
−10
5
Value
regulation of gene silencing
SIN3
CDC7
MCM5
RAP1
HDA1
UME1
BDF1
PHO23
HIR2
HIR1
SAP30
DEP1
RTF1
RXT2
CTR9
YTA7
PAF1
ESC1
CDC6
ORC5
SIF2
HST2
CTI6
YAP5
PUF4
CDC45
HHO1
WTM2
WTM1
UTH1
SDS3
ADR1
SIR4
RPD3
NAT4
FKH1
ESS1
SIR3
FKH2
OAF1
NFI1
TBF1
YRB2
Gene
Z_Shift_K_Doxo_HLD
Z_Im_K_Doxo_HLD
Z_Shift_K_Doxo_EG
Z_Im_K_Doxo_EG
Z_Shift_L_Doxo_HLD
Z_Im_L_Doxo_HLD
Z_Shift_L_Doxo_EG
Z_Im_L_Doxo_EG

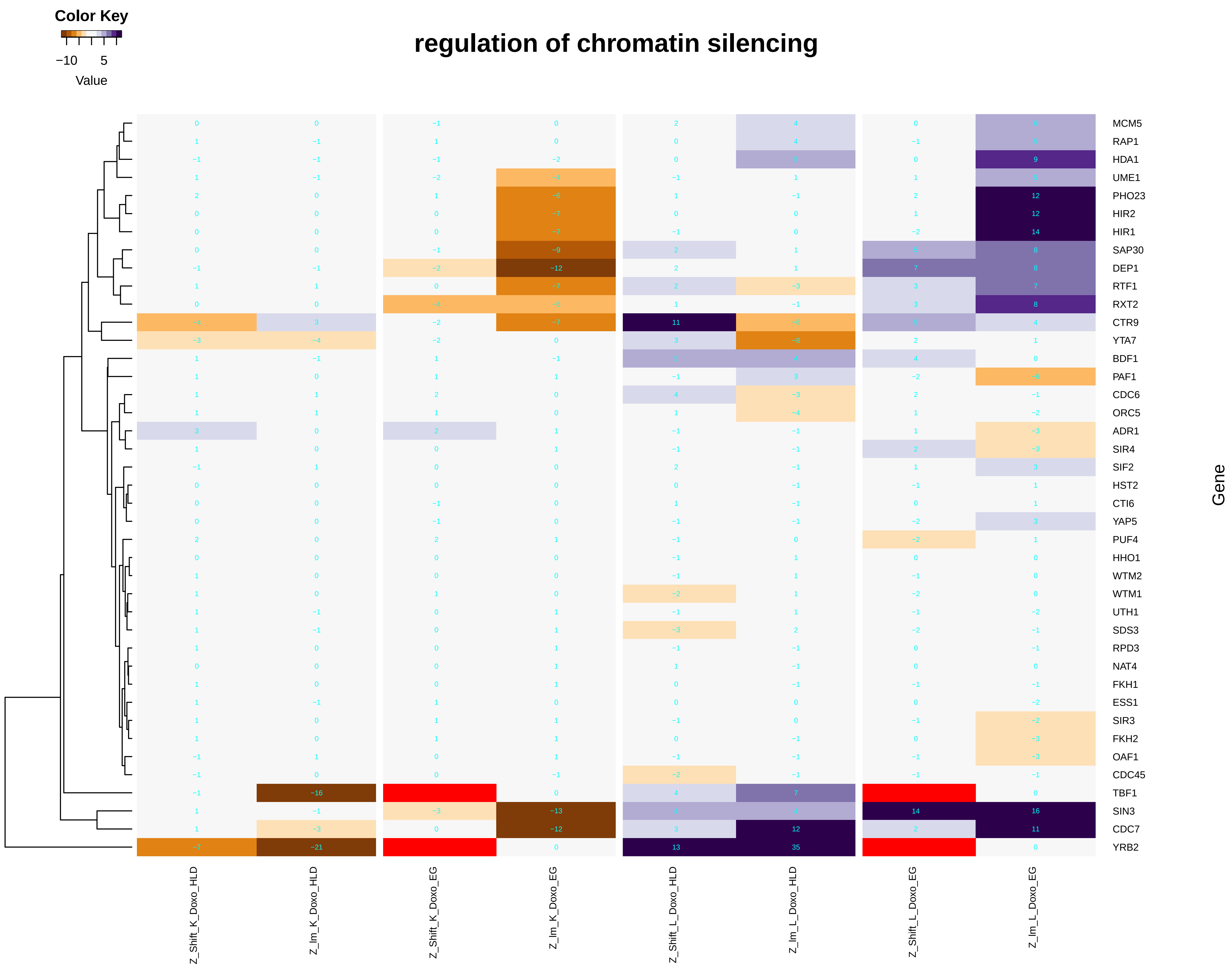
Color Key
−10
5
Value
regulation of chromatin silencing
MCM5
RAP1
HDA1
UME1
PHO23
HIR2
HIR1
SAP30
DEP1
RTF1
RXT2
CTR9
YTA7
BDF1
PAF1
CDC6
ORC5
ADR1
SIR4
SIF2
HST2
CTI6
YAP5
PUF4
HHO1
WTM2
WTM1
UTH1
SDS3
RPD3
NAT4
FKH1
ESS1
SIR3
FKH2
OAF1
CDC45
TBF1
SIN3
CDC7
YRB2
Gene
Z_Shift_K_Doxo_HLD
Z_lm_K_Doxo_HLD
Z_Shift_K_Doxo_EG
Z_lm_K_Doxo_EG
Z_Shift_L_Doxo_HLD
Z_lm_L_Doxo_HLD
Z_Shift_L_Doxo_EG
Z_lm_L_Doxo_EG

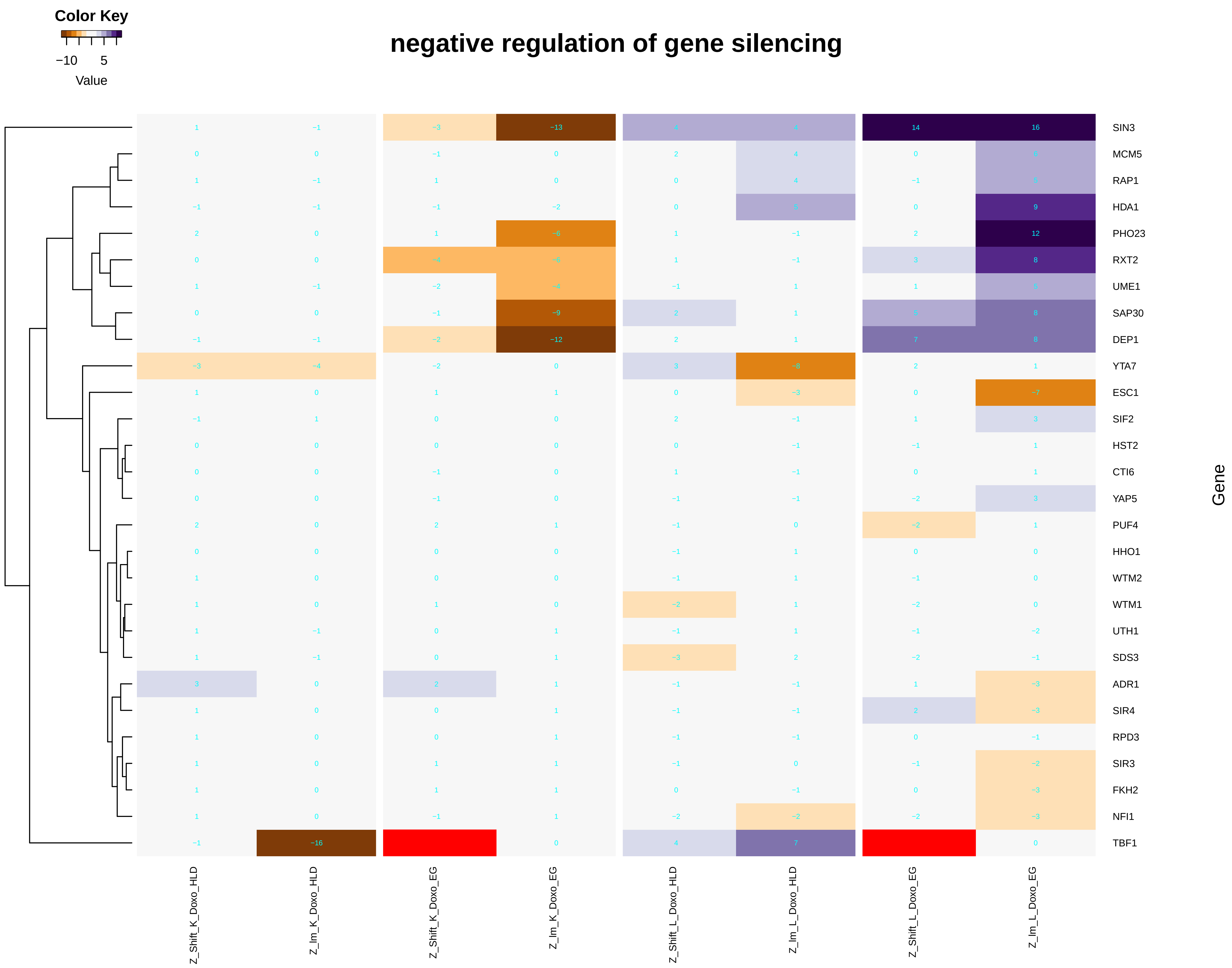

negative regulation of gene silencing
Color Key
−10
5
Value
Gene
SIN3
MCM5
RAP1
HDA1
PHO23
RXT2
UME1
SAP30
DEP1
YTA7
ESC1
SIF2
HST2
CTI6
YAP5
PUF4
HHO1
WTM2
WTM1
UTH1
SDS3
ADR1
SIR4
RPD3
SIR3
FKH2
NFI1
TBF1
Z_Shift_K_Doxo_HLD
Z_Im_K_Doxo_HLD
Z_Shift_K_Doxo_EG
Z_Im_K_Doxo_EG
Z_Shift_L_Doxo_HLD
Z_Im_L_Doxo_HLD
Z_Shift_L_Doxo_EG
Z_Im_L_Doxo_EG

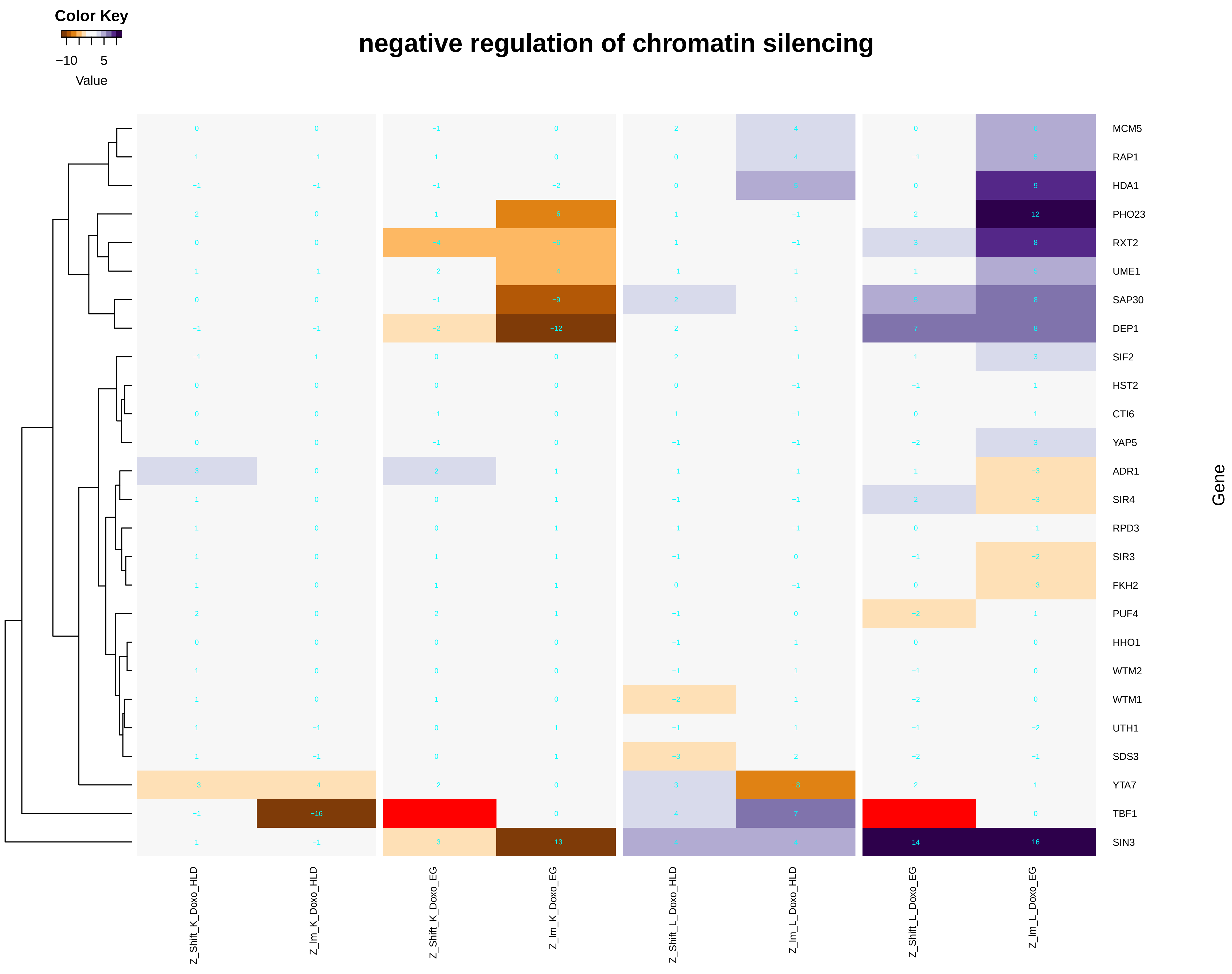
Color Key
−10
5
Value
negative regulation of chromatin silencing
MCM5
RAP1
HDA1
PHO23
RXT2
UME1
SAP30
DEP1
SIF2
HST2
CTI6
YAP5
ADR1
SIR4
RPD3
SIR3
FKH2
PUF4
HHO1
WTM2
WTM1
UTH1
SDS3
YTA7
TBF1
SIN3
Gene
Z_Shift_K_Doxo_HLD
Z_Im_K_Doxo_HLD
Z_Shift_K_Doxo_EG
Z_Im_K_Doxo_EG
Z_Shift_L_Doxo_HLD
Z_Im_L_Doxo_HLD
Z_Shift_L_Doxo_EG
Z_Im_L_Doxo_EG

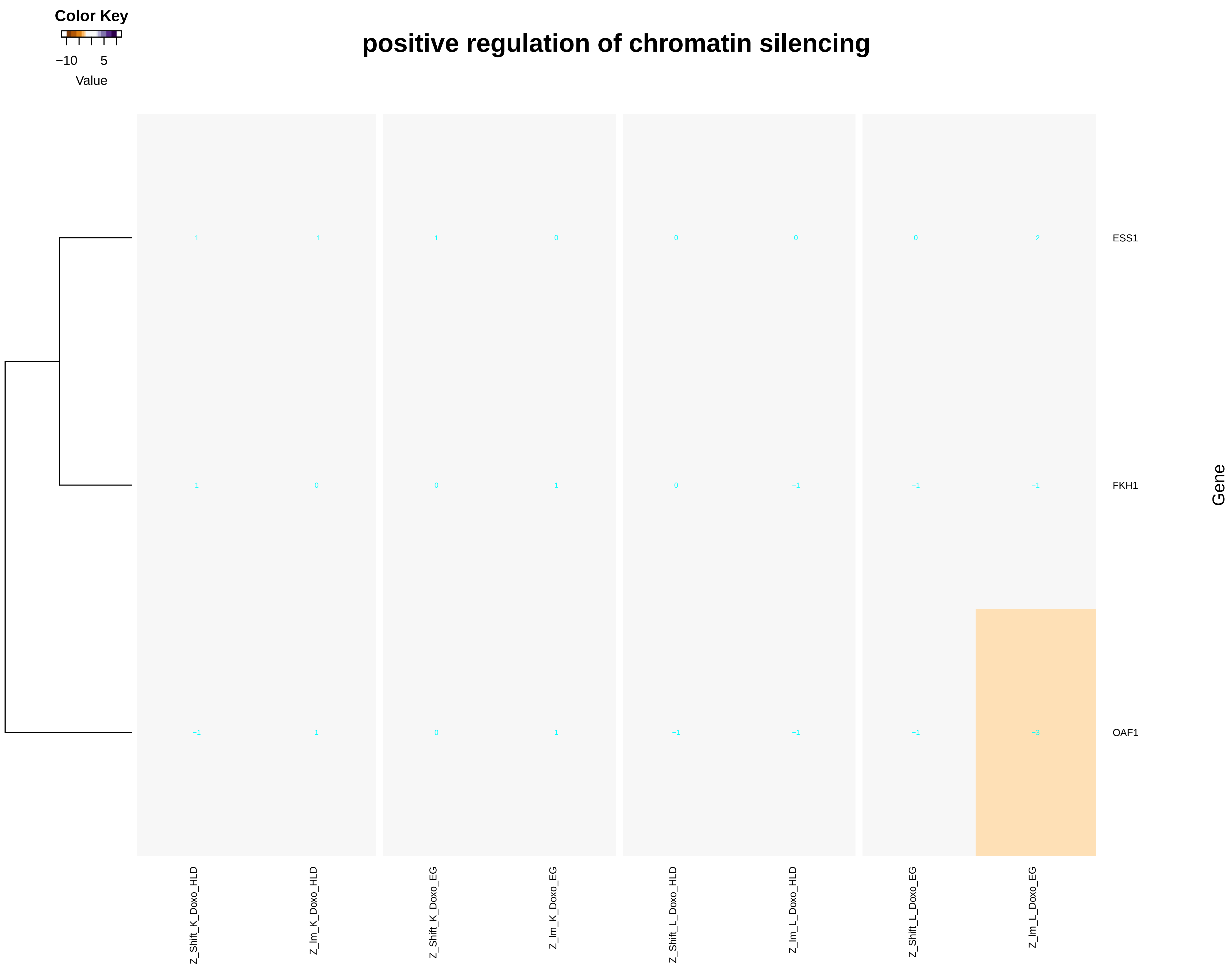

Color Key
−10
5
Value
positive regulation of chromatin silencing
ESS1
FKH1
OAF1
Gene
Z_Shift_K_Doxo_HLD
Z_Im_K_Doxo_HLD
Z_Shift_K_Doxo_EG
Z_Im_K_Doxo_EG
Z_Shift_L_Doxo_HLD
Z_Im_L_Doxo_HLD
Z_Shift_L_Doxo_EG
Z_Im_L_Doxo_EG

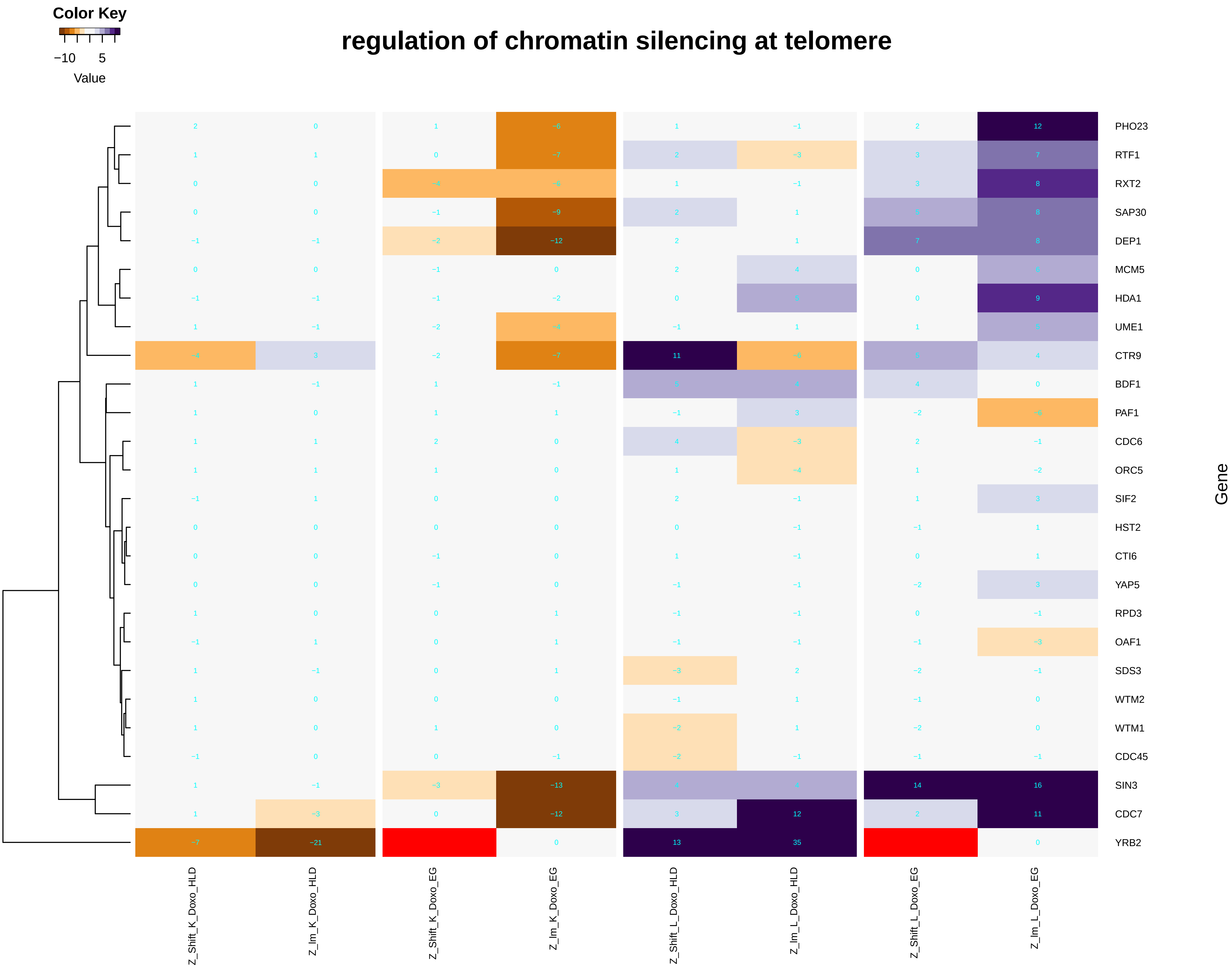
Color Key
−10
5
Value
regulation of chromatin silencing at telomere
PHO23
RTF1
RXT2
SAP30
DEP1
MCM5
HDA1
UME1
CTR9
BDF1
PAF1
CDC6
ORC5
SIF2
HST2
CTI6
YAP5
RPD3
OAF1
SDS3
WTM2
WTM1
CDC45
SIN3
CDC7
YRB2
Gene
Z_Shift_K_Doxo_HLD
Z_Im_K_Doxo_HLD
Z_Shift_K_Doxo_EG
Z_Im_K_Doxo_EG
Z_Shift_L_Doxo_HLD
Z_Im_L_Doxo_HLD
Z_Shift_L_Doxo_EG
Z_Im_L_Doxo_EG

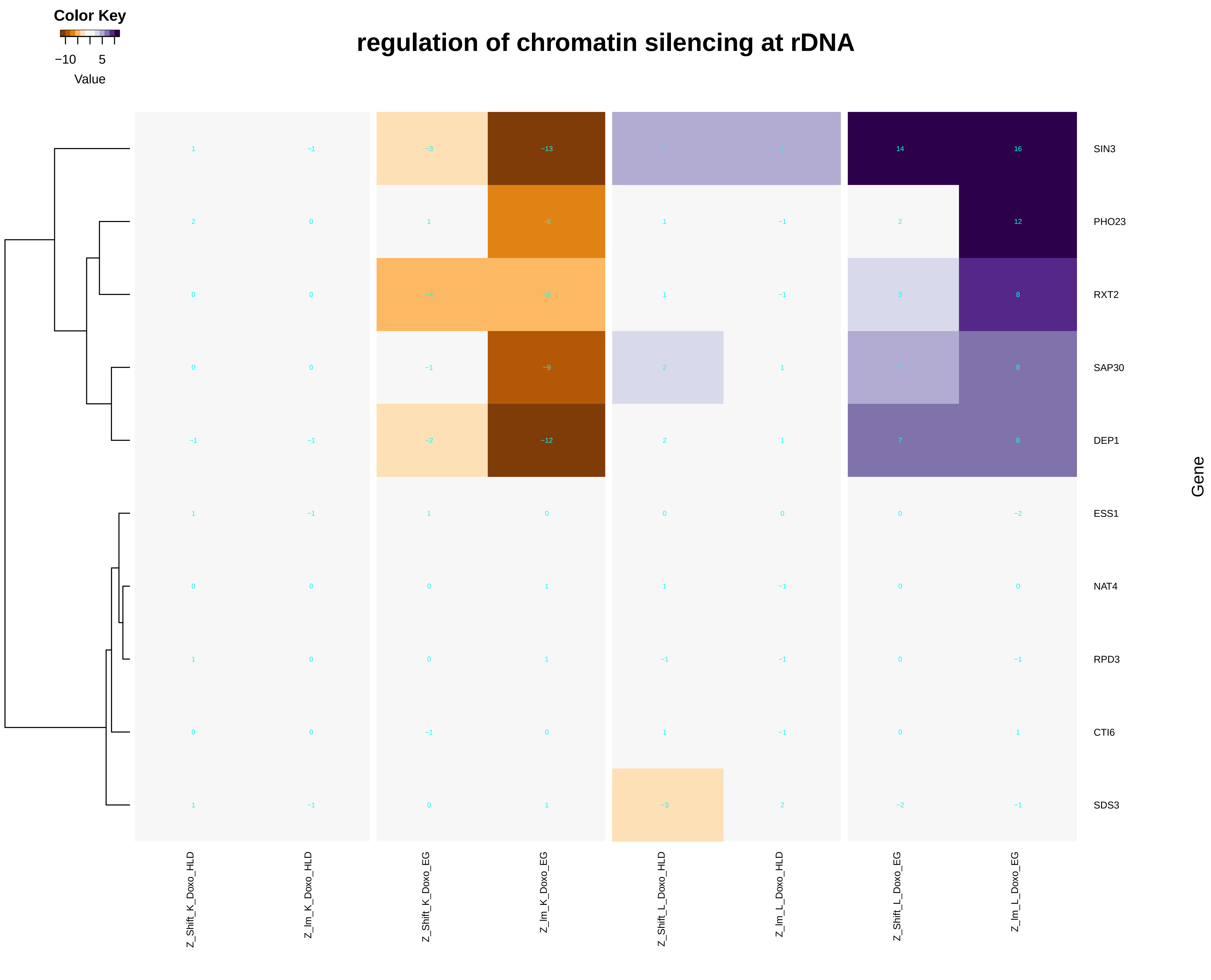

regulation of chromatin silencing at rDNA
Color Key
−10
5
Value
SIN3
PHO23
RXT2
SAP30
DEP1
ESS1
NAT4
RPD3
CTI6
SDS3
Gene
Z_Shift_K_Doxo_HLD
Z_Im_K_Doxo_HLD
Z_Shift_K_Doxo_EG
Z_Im_K_Doxo_EG
Z_Shift_L_Doxo_HLD
Z_Im_L_Doxo_HLD
Z_Shift_L_Doxo_EG
Z_Im_L_Doxo_EG

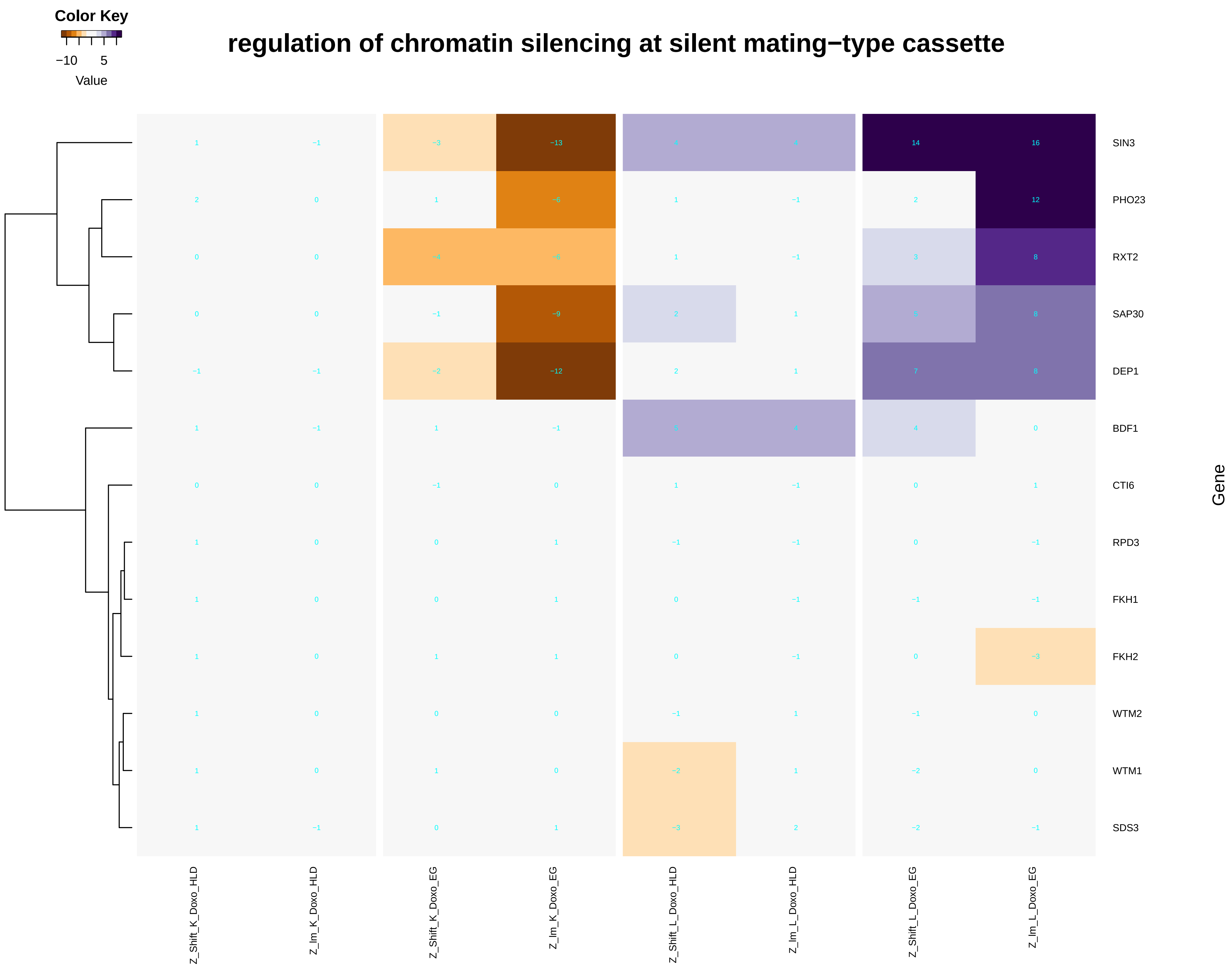
Color Key
−10
5
Value
regulation of chromatin silencing at silent mating−type cassette
SIN3
PHO23
RXT2
SAP30
DEP1
BDF1
CTI6
RPD3
FKH1
FKH2
WTM2
WTM1
SDS3
Gene
Z_Shift_K_Doxo_HLD
Z_Im_K_Doxo_HLD
Z_Shift_K_Doxo_EG
Z_Im_K_Doxo_EG
Z_Shift_L_Doxo_HLD
Z_Im_L_Doxo_HLD
Z_Shift_L_Doxo_EG
Z_Im_L_Doxo_EG

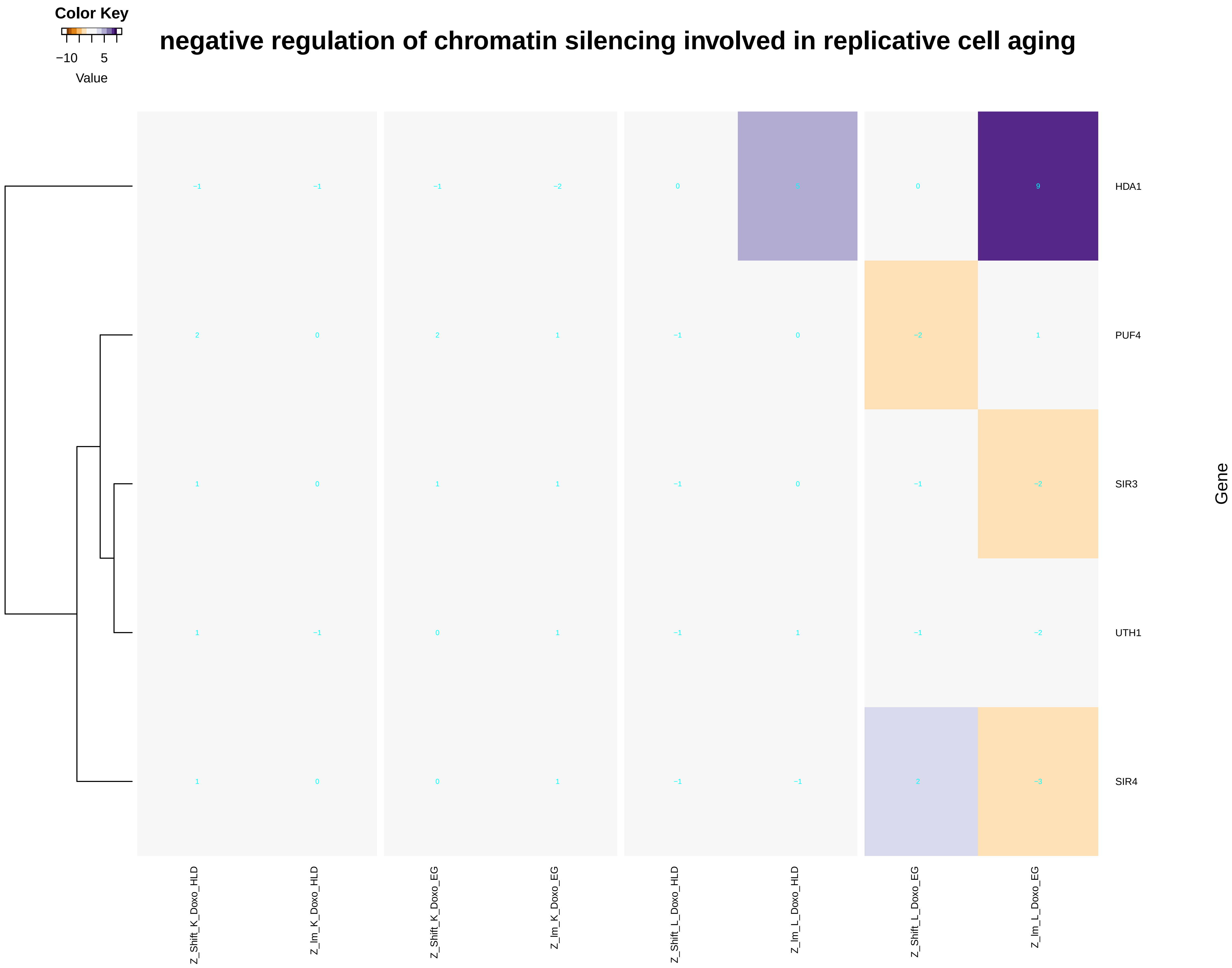

Color Key
−10
5
Value
negative regulation of chromatin silencing involved in replicative cell aging
HDA1
PUF4
SIR3
UTH1
SIR4
Gene
Z_Shift_K_Doxo_HLD
Z_lm_K_Doxo_HLD
Z_Shift_K_Doxo_EG
Z_lm_K_Doxo_EG
Z_Shift_L_Doxo_HLD
Z_lm_L_Doxo_HLD
Z_Shift_L_Doxo_EG
Z_lm_L_Doxo_EG
−1 −1 −1 −2 0 5 0 9
2 0 2 1 −1 0 −2 1
1 0 1 1 −1 0 −1 −2
1 −1 0 1 −1 1 −1 −2
1 0 0 1 −1 −1 2 −3

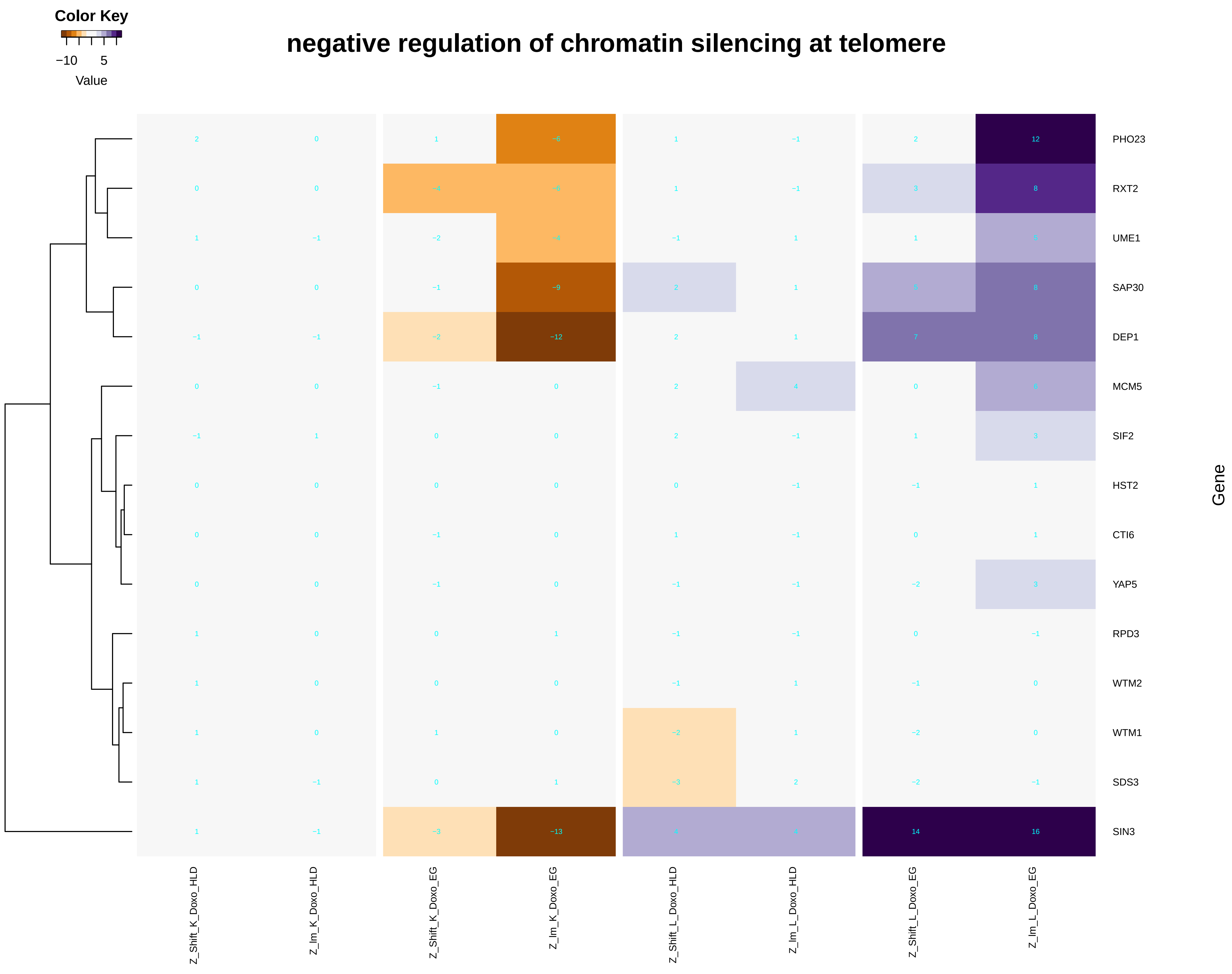
Color Key
−10
5
Value
negative regulation of chromatin silencing at telomere
PHO23
RXT2
UME1
SAP30
DEP1
MCM5
SIF2
HST2
CTI6
YAP5
RPD3
WTM2
WTM1
SDS3
SIN3
Gene
Z_Shift_K_Doxo_HLD
Z_lm_K_Doxo_HLD
Z_Shift_K_Doxo_EG
Z_lm_K_Doxo_EG
Z_Shift_L_Doxo_HLD
Z_lm_L_Doxo_HLD
Z_Shift_L_Doxo_EG
Z_lm_L_Doxo_EG

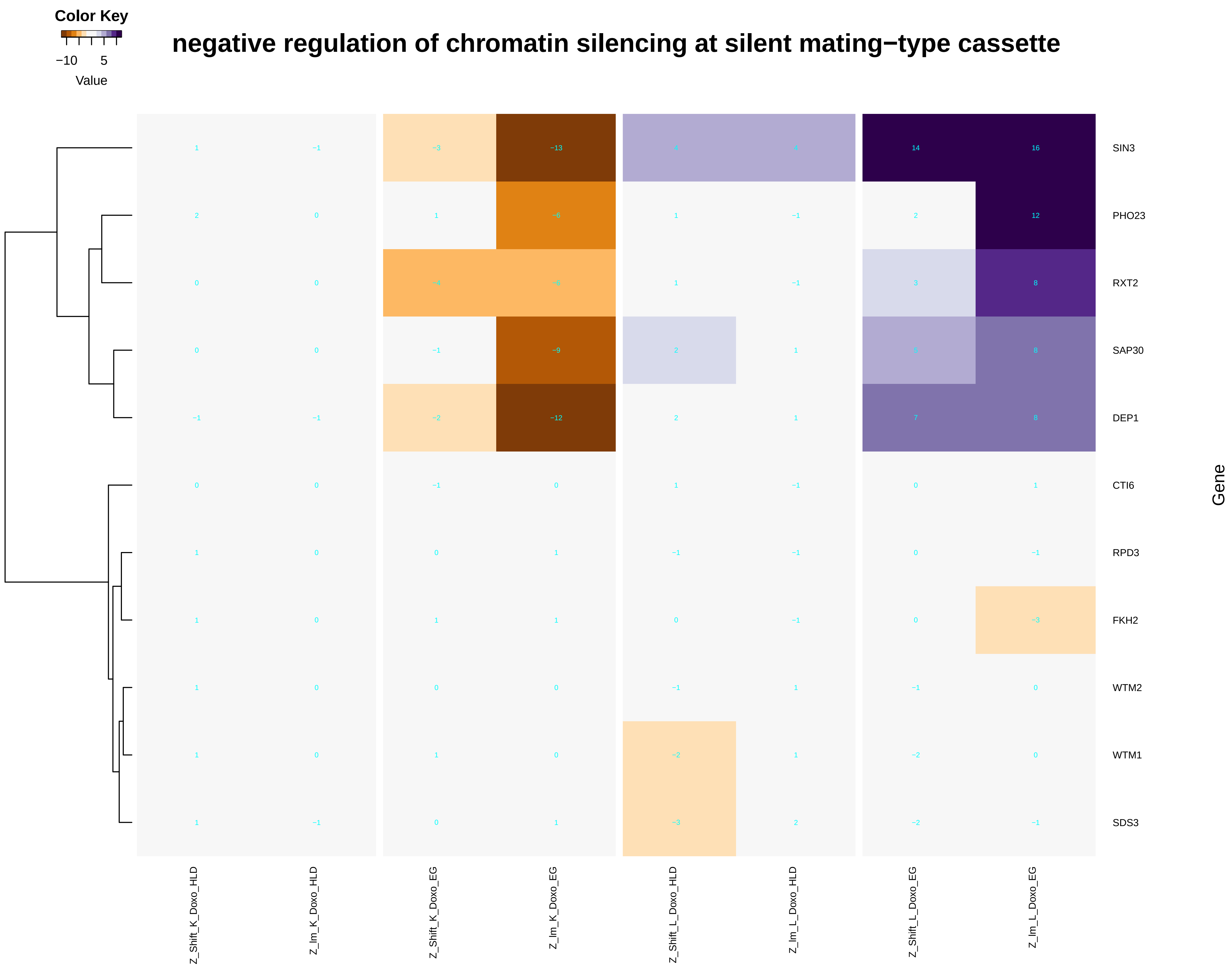
negative regulation of chromatin silencing at silent mating−type cassette
Color Key
−10
5
Value
SIN3
PHO23
RXT2
SAP30
DEP1
CTI6
RPD3
FKH2
WTM2
WTM1
SDS3
Gene
Z_Shift_K_Doxo_HLD
Z_lm_K_Doxo_HLD
Z_Shift_K_Doxo_EG
Z_lm_K_Doxo_EG
Z_Shift_L_Doxo_HLD
Z_lm_L_Doxo_HLD
Z_Shift_L_Doxo_EG
Z_lm_L_Doxo_EG

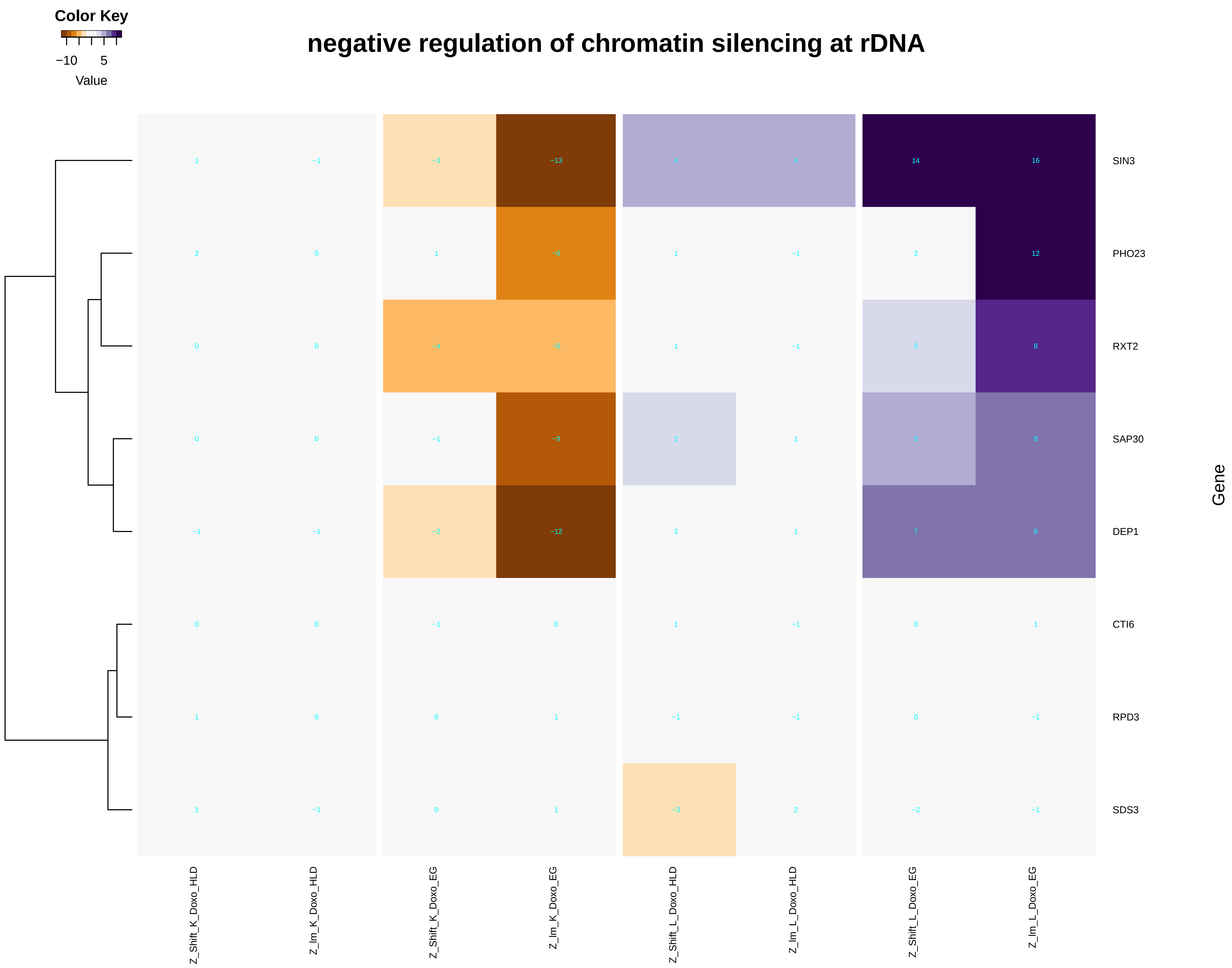
Color Key
−10
5
Value
negative regulation of chromatin silencing at rDNA
SIN3
PHO23
RXT2
SAP30
DEP1
CTI6
RPD3
SDS3
Gene
Z_Shift_K_Doxo_HLD
Z_Im_K_Doxo_HLD
Z_Shift_K_Doxo_EG
Z_Im_K_Doxo_EG
Z_Shift_L_Doxo_HLD
Z_Im_L_Doxo_HLD
Z_Shift_L_Doxo_EG
Z_Im_L_Doxo_EG
